# The small FNR regulon of *Neisseria gonorrhoeae*: comparison with the larger *Escherichia coli *FNR regulon and interaction with the NarQ-NarP regulon

**DOI:** 10.1186/1471-2164-8-35

**Published:** 2007-01-29

**Authors:** Rebekah N Whitehead, Tim W Overton, Lori AS Snyder, Simon J McGowan, Harry Smith, Jeff A Cole, Nigel J Saunders

**Affiliations:** 1 School of Biosciences, University of Birmingham, Edgbaston, Birmingham B15 2TT, UK; 2 The Bacterial Pathogenesis and Functional Genomics Group, The Sir William Dunn School of Pathology, University of Oxford, South Parks Road, Oxford, OX1 3RE, UK; 3 The Computational Biology Research Group, The Sir William Dunn School of Pathology, University of Oxford, South Parks Road, Oxford, OX1 3RE, UK; 4 The Medical School, The University of Birmingham, Edgbaston, Birmingham B15 2TT, UK

## Abstract

**Background:**

*Neisseria gonorrhoeae *can survive during oxygen starvation by reducing nitrite to nitrous oxide catalysed by the nitrite and nitric oxide reductases, AniA and NorB. The oxygen-sensing transcription factor, FNR, is essential for transcription activation at the *aniA *promoter, and full activation also requires the two-component regulatory system, NarQ-NarP, and the presence of nitrite. The only other gene known to be activated by the gonococcal FNR is *ccp *encoding a cytochrome *c *peroxidase, and no FNR-repressed genes have been reported in the gonococcus. In contrast, FNR acts as both an activator and repressor involved in the control of more than 100 operons in *E. coli *regulating major changes in the adaptation from aerobic to anaerobic conditions. In this study we have performed a microarray-led investigation of the FNR-mediated responses in *N. gonorrhoeae *to determine the physiological similarities and differences in the role of FNR in cellular regulation in this species.

**Results:**

Microarray experiments show that *N. gonorrhoeae *FNR controls a much smaller regulon than its *E. coli *counterpart; it activates transcription of *aniA *and thirteen other genes, and represses transcription of six genes that include *dnrN *and *norB*. Having previously shown that a single amino acid substitution is sufficient to enable the gonococcal FNR to complement an *E. coli fnr *mutation, we investigated whether the gonococcal NarQ-NarP can substitute for *E. coli *NarX-NarL or NarQ-NarP. A plasmid expressing gonococcal *narQ-narP *was unable to complement *E. coli narQP *or *narXL *mutants, and was insensitive to nitrate or nitrite. Mutations that progressively changed the periplasmic nitrate sensing region, the P box, of *E. coli *NarQ to the sequence of the corresponding region of gonococcal NarQ resulted in loss of transcription activation in response to the availability of either nitrate or nitrite. However, the previously reported ligand-insensitive ability of gonococcal NarQ, the "locked on" phenotype, to activate either *E. coli *NarL or NarP was confirmed.

**Conclusion:**

Despite the sequence similarities between transcription activators of *E. coli *and *N. gonorrhoeae*, these results emphasise the fundamental differences in transcription regulation between these two types of pathogenic bacteria.

## Background

*Neisseria gonorrhoeae *is an obligate human pathogen with no known environmental reservoirs. It can be isolated from gonorrhoea patients in clinical samples in which obligately anaerobic bacteria are abundant [1,2], suggesting that gonococci encounter and survive without oxygen in their natural habitat. Clark and her colleagues have shown that gonococci can grow anaerobically using a truncated denitrification pathway in which nitrite is reduced to nitrous oxide, catalysed by the copper-containing nitrite reductase, AniA, and the single subunit nitric oxide reductase, NorB [2-5]. Nitrite reduction is severely repressed by oxygen, but is induced during anaerobic growth by the global transcription factor, FNR (for regulator of fumarate and nitrate reduction), and by a two-component regulatory system that we designated NarQ-NarP [5,6]).

Until complete genome sequences became available in the last ten years, it was commonly assumed that obligate pathogens rely less on transcription control than more versatile bacteria that occupy a variety of niches outside of their mammalian hosts. With reference to *N. gonorrhoeae*, this impression was reinforced by the fact that *ccp *(encoding a cytochrome *c *peroxidase [5,7]) is the only gene other than *aniA *that is known to be regulated by the gonococcal FNR, and by the widespread distribution of repeat DNA sequences that promote a high frequency of genetic variation-based expression control resulting in gene silencing, phase variation, and antigenic shift. The availability of the complete genome sequence and pan-*Neisseria *microarrays provide an opportunity to test these assumptions directly by comparing the extent of the *N. gonorrhoeae *FNR regulon with that of the recently-published *E. coli *FNR regulon [8]. As full expression of *aniA *in the gonococcus and both of the major nitrite reductases in *E. coli *all require a functional two-component regulatory system (NarQP in the gonococcus; both NarQP and NarXL in *E. coli*), we have also investigated the similarities and differences between the NarQP systems of these bacteria.

## Results

### Microarray analysis of the gonococcal FNR regulon

**Figure 1:**
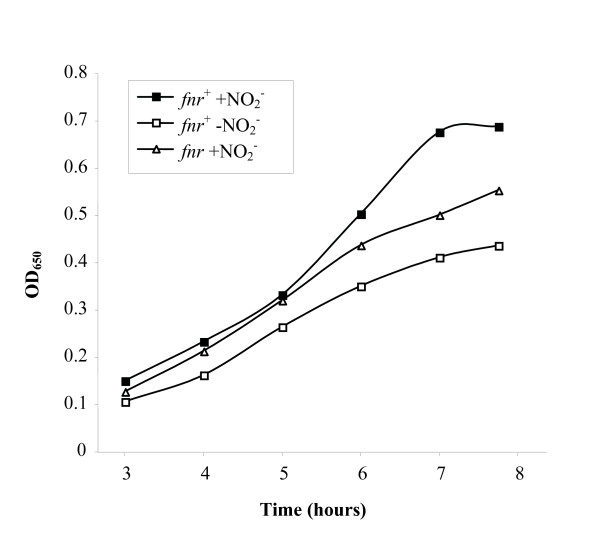
Growth characteristics of strain RUG7001 (*fnr*^+^) in the presence and absence of 5 mM sodium nitrite and strain RUG7002 (*fnr*) in the presence of nitrite. Since expression of the nitrite reductase AniA is dependent upon FNR, the *fnr *strain cannot respire nitrite.

To determine the range of functions regulated by FNR in *N. gonorrhoeae*, the *fnr*^+ ^parental strain, RUG7001, and its isogenic *fnr *mutant, RUG7022, were grown in poorly aerated cultures until oxygen became limiting and *aniA *expression had been induced (as indicated by the disappearance of nitrite from the medium). The *fnr*^+ ^parental strain grew exponentially until the nitrite had been reduced. In contrast, the *fnr *mutant grew at a constant rather than an exponential rate that was similar to the oxygen-limited growth of the parental strain in the absence of nitrite (Fig. 1). To distinguish between genes differentially expressed in response to growth rate from those regulated directly by FNR in response to oxygen limitation, RNA was extracted from the *fnr*^+ ^parental strain grown in the presence or absence of nitrite, and from the *fnr *mutant grown in the presence of nitrite. These RNA preparations were analysed using two pair wise comparisons: *fnr*^+ ^gonococci growing exponentially in the presence of nitrite compared to the *fnr *mutant; and *fnr*^+ ^gonococci growing in the absence of nitrite compared to the *fnr *mutant. Data were analysed according to fold-change (> 2-fold difference between the two strains) and a Student's t-test, using a cut-off *p *value of 0.01, reflecting the fact that six biological replicates were used in this experiment. Fourteen transcripts were more abundant in the parental strain than the *fnr *mutant, while six were more abundant in the mutant, suggesting FNR repression (Table 1). Five of the transcripts were more abundant in the *fnr*^+ ^strain during growth in both the presence and absence of nitrite, suggesting direct FNR regulation. The most up-regulated transcripts encode a short transcript of unknown function (46.3-fold, *p *1.1 × 10^-5 ^with nitrite; 73.06-fold, *p *7.3 × 10^-6 ^without nitrite), and a putative iron uptake outer membrane protein designated OmpU (NGO1688; 12.27-fold, *p *3.2 × 10^-4 ^with nitrite; 6.6-fold, *p *7.3 × 10^-5 ^without nitrite) [[9]; see also comments in the Entrez nucleotide entry for *N. meningitidis ompU*, accession AF118122]. Also activated were the nitrite reductase gene, *aniA *(6.02-fold, *p *1.9 × 10^-5 ^with nitrite; 3.56-fold, *p *8.2 × 10^-4 ^without nitrite), NGO1215 encoding a highly conserved hypothetical protein (4.57-fold, *p *4.8 × 10^-4 ^with nitrite; 3.52-fold, *p *3.2 × 10^-5 ^without nitrite); and NGO0546 encoding the Res subunit of a Type III restriction-modification system similar to the *Eco*PI enzyme (2.31-fold, *p *2.1 × 10^-3 ^with nitrite; 2.88-fold, *p *5.2 × 10^-5 ^without nitrite). Nine further transcripts were more highly expressed in the *fnr*^+ ^strain compared to the *fnr *mutant, but only during growth in the presence of nitrite. These transcripts are possibly regulated in response to growth rate rather than by FNR. Only one of these genes, NGO0602, encoding a putative MerR-family transcription regulator, was also more highly expressed in the *fnr*^+ ^strain grown in the absence of nitrite (2.8-fold), but the regulation of this gene was not as statistically significant (*p *0.05).

Most down-regulated by FNR was the NGO1716 transcript encoding a putative phosphotransferase (COG3178; 0.14-fold, *p *9.6 × 10^-6 ^with nitrite; 0.18-fold, *p *8.1 × 10^-6 ^without nitrite), followed by two genes implicated in nitric oxide metabolism, *dnrN *(0.37-fold, *p *0.12 with nitrite; 0.19-fold, *p *8.4 × 10^-6 ^without nitrite), and *norB *(0.6-fold, *p *0.84 with nitrite; 0.28-fold, *p *2.9 × 10^-3 ^without nitrite). Two transcripts were less abundant in the parental strain during growth in the presence of nitrite: cysteine synthetase (*cysK *gene; NGO0340; 0.29-fold, *p *7.3 × 10^-3^); and *glnQ *(NGO0374) encoding a component of an ABC-type amino acid transporter (0.47-fold; *p *3.4 × 10^-4^). The transcript for a *Neisseria*-specific protein encoded by NGO1428 was more abundant in the mutant only in the absence of nitrite (0.49-fold, *p *2.6 × 10^-4^). These data are summarized in Table 1.

The results of the microarray experiments can be interactively interrogated in an on-line graphical GBrowse database at , where the fold ratio, number of observations for each gene, Student's t-test, Cyber-T *p*-values, XNG and NGO annotations, and the microarray probe locations can be visualized, and searched using chromosomal locations, gene names, or gene identifiers. The results from each experiment can be viewed individually or in combination to compare the results, and users can add their own local annotations. The results of these experiments can also be seen in direct comparison with the expression data obtained in a previous microarray study addressing expression associated with *narPQ *[10].

### Bioinformatic and ChIP analysis of promoters of genes differentially expressed in the fnr mutant

Putative FNR binding sites with at least a 7/10 match to the *E. coli *consensus, TTGATNNNNATCAA, were identified within 200 base pairs upstream of the translation start codon of eleven of the genes revealed by the microarray experiments to be differentially expressed in the *fnr *mutant and its parent (Table 1). A strain containing a chromosomal *fnr*-3xFLAG fusion was constructed (*N. gonorrhoeae *strain JCGC502), grown microaerobically both in the presence and absence of nitrite to the late exponential phase, DNA-binding proteins were cross linked to the chromosome, the bacteria were lysed and chromosomal DNA was sheared. Anti-FLAG antibodies were used to immunoprecipitate FNR-DNA complexes, which were de-crosslinked and the DNA released was purified. The quantity of each promoter fragment in the immunoprecipitated DNA pool was measured by realtime PCR, relative to the FNR-independent *hpt *promoter. Promoter fragments enriched 60% or more in at least two independent experiments scored positive (Table 1).

Only one of the fourteen genes potentially activated by FNR had previously been reported to be FNR-dependent: *aniA*, encoding a nitrite reductase [5,6]. Consistent with the microarray data reported above, FNR binding to P_*aniA *_was confirmed by ChIP, providing a positive control for the ChIP data. The ChIP experiments also confirmed FNR-binding to the promoter regions of the most highly FNR-activated transcript (NMB1205) and *ompU*. Although the *ompU *promoter has no recognisable FNR binding site, multiple potential half-sites are present in the promoter region. Conversely, even though open reading frames NGO1215 and NGO0546 have potential FNR binding sites upstream, FNR binding was not detected by ChIP. This emphasises that caution is required when drawing conclusions from data based upon either of these techniques alone.

### Proteomic analysis of the FNR regulon

A study of protein expression in *fnr*^+ ^and *fnr *gonococci revealed fewer differences than were identified in the microarray study. Comparison of the cytoplasmic proteins of the *fnr*^+ ^parental strain, RUG7001, and the *fnr *mutant, strain RUG7022, grown in the presence of nitrite revealed no significant differences in protein expression (data not shown). In contrast, three membrane proteins were more abundant in the *fnr*^+ ^strain: nitrite reductase, AniA; nitric oxide reductase, NorB; and the septum site-determining cell division protein, MinD [11]. Although *norB *transcription is independent of FNR [12], more NorB accumulated during nitrite reduction by the *fnr*^+ ^strain than in the *fnr *mutant. The explanation for this apparent contradiction is that transcription from the *norB *promoter is induced by NO [9,12], so because AniA is not synthesised in the mutant, no NO would be generated to activate the expression of *norB*. As the microarray data did not identify *minD *expression to be activated by FNR, the apparent differential expression of MinD protein, revealed by proteomic analysis, is more likely to be due to a growth rate effect than to a direct effect of FNR.

### Effects of iron deprivation and peroxide stress on an fnr mutant

In light of the altered expression of the genes associated with iron transport (*ompU *and the putative NRAMP family member NGO1455) it was determined whether FNR is important in metal ion uptake. The ability of a gonococcal *fnr *mutant to survive metal ion limitation was tested. Microaerobically grown *fnr *mutant and parent were treated with 200 μM dipyridyl, a chelator of iron and manganese ions. Viable counts made at regular intervals for up to one hour showed there was no significant difference in survival between the *fnr*^+ ^and *fnr *gonococci.

Considering that genes predicted to be involved in defence against ROS (*ccp *[7]) or induced upon exposure to ROS (NGO1428 and NGO1716 [13]) were observed to be regulated by FNR, the ability of an *fnr *mutant to survive oxidative stress was tested. Oxygen-limited cultures of gonococci, both the *fnr *mutant and the parental strain, were subjected to 10 mM hydrogen peroxide. Viable counts taken at intervals up to one hour showed, as with the metal chelation experiment, no significant difference in survival between the *fnr*^+ ^and *fnr *bacteria.

### Why cytochrome *c *peroxidase was not identified as being FNR-regulated by microarray analysis

Expression of the cytochrome *c *peroxidase is activated by FNR in response to oxygen [5]. Mature CCP protein was detected by staining gonococcal whole cell or membrane proteins separated by SDS-PAGE for haem-dependent peroxidase activity. However, in the present microarray study, FNR-dependent expression of the *ccp *gene was not observed because of the low level of expression of *ccp*. The quantity of *ccp *transcript was not sufficient to generate a statistically significant signal above the background signal of the slide, and was filtered out at the pON filter stage of data analysis.

**Figure 2:**
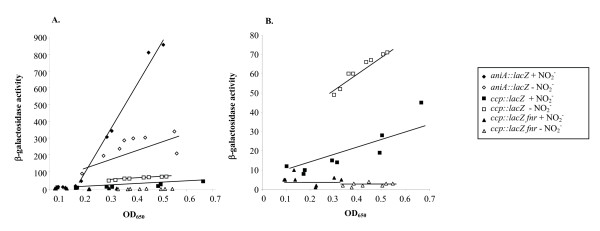
A. β-galactosidase activity of the *aniA*::*lacZ *and *ccp*::*lacZ *fusions. Strains RUG7001 (*aniA*::*lacZ*), JCGC201 (*ccp*::*lacZ*), and JCGC202 (*ccp*::*lacZ fnr*) were grown in liquid culture with or without 5 mM NaNO_*2*_. Samples were taken at hourly intervals, β-galactosidase activity measured, and the β-galactosidase activities plotted against optical density at 650 nm. B. Data for the JCGC201 (*ccp*::*lacZ*) and JCGC202 (*ccp*::*lacZ fnr*) fusions are shown on an expanded scale to illustrate more clearly the effects of FNR and nitrite on *ccp *expression.

To confirm that the *ccp *gene is regulated by FNR, and to compare the relative activity of the *ccp *and *aniA *promoters, *N. gonorrhoeae *strains RUG7001, carrying a chromosomal *aniA::lacZ *fusion, and JCGC201 & JCGC202, *fnr*^+ ^and *fnr *derivatives carrying *ccp::lacZ*, were grown microaerobically in gonococcal broth (GCB) in the presence and absence of nitrite and assayed for β-galactosidase activity after 4, 5, 6, and 7 hours of growth (Figure 2). Far less β-galactosidase activity accumulated in the *ccp*::*lacZ fnr *mutant strain than in the *fnr*^+ ^strain, and the β-galactosidase activity of the *fnr*^+ ^strain was higher in the absence than in the presence of nitrite. During growth in the absence of nitrite, at an OD_650 _of around 0.5 (corresponding to 0.2 g bacterial dry weight l^-1^), FNR activated P_*ccp *_24-fold^. ^These data support the previously reported observation that *ccp *expression is repressed by the presence of nitrite in an *fnr*^+ ^strain [7]. Furthermore, expression of *ccp *was observed to be FNR-dependent in quantitative real time PCR experiments in which transcript levels in *fnr *mutant and wild-type strains were compared. Whereas in the parental strain *ccp *expression was induced 18-fold during oxygen limited growth, there was no induction of *ccp *expression in the *fnr *mutant strain. These data confirm that, while the *ccp *gene is not included in the list of FNR-activated genes from the microarray data due to low expression levels, the *ccp *promoter is activated by FNR. In addition, the *ccp *promoter was enriched in ChIP experiments, so was shown to bind FNR *in vivo*. However, these control *ccp *experiments illustrate a rarely documented weakness of microarray experiments, namely the problem of false negative results that, due to over-stringent use of statistical analysis, might lead to under-estimation of the number of differentially expressed genes. It also illustrates the utility of an analytical approach that incorporates exclusion of data for transcripts that do not generate detectable signals; so that those genes below the detectable thresholds are readily identified, rather than using 'flooring' or other methods for filling in low intensity or empty microarray data fields.

### The gonococcal NarQP cannot complement an *E. coli narQP *mutation

The microarray data revealed fundamental differences between the FNR regulons of *E. coli *and *N. gonorrhoeae*, not least in that the gonococcal FNR regulon is very small compared with its *E. coli *counterpart. However, in both organisms, expression of the major nitrite reductases is co-activated by FNR and a two-component regulatory system, NarQ-NarP, that bind to similar target sequences located at almost identical positions relative to the respective transcription start sites (P_*aniA *_in *N. gonorrhoeae*; P_*nirB *_in *E. coli *[5,6,14-18]). We have previously demonstrated that a single amino acid substitution is sufficient to enable the gonococcal FNR to complement an *E. coli fnr *mutation. It was therefore of interest to determine whether the gonococcal NarQ-NarP could complement *E. coli *mutants defective in both NarXL and NarQP.

The gonococcal *narQP *genes were expressed in *E. coli *under the control of the *E. coli fnr *promoter from plasmid pGCNarQP, and the ability of the gonococcal NarQP proteins to activate the *E. coli nirB *promoter was assessed [19]. Expression of the *E. coli *nitrite reductase NirBD is activated by FNR in response to oxygen limitation and by NarQP in response to the availability of nitrate or nitrite [20]. *E. coli *strains JCB386 (*nirB*::*lacZ*), JCB3861 (*nirB*::*lacZ narXL*), JBC3863 (*nirB*::*lacZ narXLQP*), and JCB3863 transformed with pGCNarQP were grown anaerobically in the presence or absence of 5 mM NaNO_2 _or 20 mM NaNO_3 _and β-galactosidase activities measured (Table 2A).

In *E. coli *strain JCB386, the *nirB *promoter is activated in response to both nitrite and nitrate. Activation in the presence of nitrite is reduced in strain JCB3861, which lacks NarXL. In the *narXLQP *strain JCB3863, *nirB *activity is very low in all three growth conditions, since NarQP is required for activation in the presence of nitrite or nitrate. When transformed with pGCNarQP, strain JCB3863 has similarly low β-galactosidase activities in all three conditions. Expression of the gonococcal *narQP *genes from pGCNarQP was verified by RT-PCR (data not shown). Therefore, it was concluded that gonococcal NarQP cannot complement an *E. coli narXLQP *mutation at P_*nirB*_.

### Ligand sensing and signal transduction characteristics of the gonococcal NarQ

Only a very limited range of genetic techniques are available to investigate sensor kinases and response regulators by site-directed mutagenesis and gene deletions in the gonococcus. However, as some sensor kinases have been shown to phosphorylate response regulators of a heterologous host [21,22], the ability of gonococcal NarQ to phosphorylate the *E. coli *NarP protein was assessed to investigate the ligand sensing and signal transduction characteristics of the gonococcal NarQ and NarP proteins. Strain JCB391 (*narXL narQ*) and JCB391 transformed with pBADgcQ, expressing gonococcal *narQ*, each co-transformed with pRNW15 carrying *napF*::*lacZ*, were grown anaerobically in the presence or absence of nitrate and nitrite and their β-galactosidase activities determined (Table 2B). The NarP-dependent *napF *promoter was not activated during growth in the presence of nitrite and nitrate in strain JCB391, since the NarQ sensor kinase was not present and NarP was unable to become phosphorylated, but was activated constitutively in strain JCB391 expressing the gonococcal NarQ from pBADgcQ. The explanation for this observation is that the gonococcal NarQ was constitutively phosphorylating the *E. coli *NarP protein, which was activating transcription. This was the first indication that the gonococcal NarQ sensor kinase might be ligand-insensitive and constitutively active in *E. coli*.

Expression of the *E. coli *fumarate reductase operon, *frdABCD*, is activated by FNR in response to oxygen limitation but repressed by NarL in response to the availability of nitrate or nitrite [23]. Due to the absence of a 7-2-7 inverted repeat sequence (where the 7 bases are the NarL heptamer), NarP is unable to bind at this promoter [24]. If the gonococcal NarQ is constitutively active, it should also be able to activate *E. coli *NarL and hence repress transcription at P_*frd*_. Strain JCB12 (*frdA::lacZ narXQ*) and strain JCB12 transformed with pBADgcQ expressing gonococcal NarQ, were grown anaerobically in the presence or absence of nitrate and nitrite and their β-galactosidase activities were determined (Table 2C). The *frdA *promoter was activated in all three conditions in strain JCB12, but was repressed in all three conditions by strain JCB12 expressing the gonococcal NarQ from pBADgcQ, as expected if the gonococcal NarQ was continually phosphorylating the *E. coli *NarL protein and therefore constitutively active in *E. coli*.

### Mutations in the P-box of *E. coli *NarQ do not alter ligand specificity

Previous studies have revealed residues in a periplasmic region of the *E. coli *NarQ and NarX proteins, the P-box, that are important for ligand sensing and discrimination between nitrate and nitrite [25-28]. Only ten of the 18 residues that comprise the gonococcal P-box are the same as those of either the *E. coli *NarQ or NarX proteins. Four residues are proposed to be important for ligand discrimination in *E. coli *NarX (H45, K49, R54 and R59); substitutions at any of these residues result in NarX proteins with altered ligand sensing characteristics [28]. Of these four, only R59 is conserved in the gonococcal NarQ. The residue in gonococcal NarQ corresponding to R54 is a lysine; an R54K substitution in *E. coli *NarX results in a ligand-insensitive phenotype. To determine whether the differences in the P-box of gonococcal NarQ determined nitrite specificity, the *E. coli *NarQ P-box was mutated to resemble the gonococcal P-box (Table 3). Mutated NarQ proteins were expressed in *E. coli *strains mutated in *narX *and *narQ *and their ability to activate *nirB*::*lacZ *or repress *frdA*::*lacZ *chromosomal fusions was assessed using β-galactosidase assays (Table 3). Substitutions in both single and multiple residues resulted in NarQ proteins that were inactive and ligand insensitive, a "locked-off" phenotype.

## Discussion

### Contrasts between the *N. gonorrhoeae *and *E. coli *FNR and NarP regulons

The first conclusion from this study is that the *N. gonorrhoeae *FNR and NarP regulons are both much smaller than their *E. coli *counterparts. Constantinidou *et al*. [8] estimated that at least 104, and possibly a many as 115, *E. coli *operons are regulated directly by FNR, including 68 that are induced, and 36 that are repressed: the FNR regulon of the pathogenic *E. coli *O157 is of a similar size (Overton, Constantinidou and Cole, unpublished data). The corresponding figures for the gonococcus are that at most 14 transcripts are induced by FNR, and 6 are repressed. Based upon DNA sequence analysis and ChIP experiments, even this might be an over-estimate of the genes that are directly regulated by FNR. However, it is similar to the 9 transcription units recently proposed to be activated by FNR in the closely related pathogen, *Neisseria meningitidis *[29]. These authors derived a consensus sequence for meningococcal FNR-binding sites that differed at one position from the consensus FNR-binding site in *E. coli*. However, three lines of evidence indicate that the consensus gonococcal FNR binding site is identical to that of *E. coli *FNR: (i) there is a perfect match to the *E. coli *consensus sequence in the regulatory region of the *N. gonorrhoeae *transcript that is most dependent upon FNR; (ii) Overton *et al*. [14] showed that a single amino acid substitution, S18F in the N-terminal domain, which is located well away from the DNA recognition helix, enables the gonococcal FNR to activate a range of FNR-dependent promoters as effectively as the *E. coli *FNR, suggesting that they have similar site specificities; and (iii) unsubstituted gonococcal FNR can function as a repressor at *E. coli *FNR-binding sites, again suggesting that they have similar, or even identical, specificities [14].

The *ccp *promoter was not identified in the microarray study to be part of the FNR regulon, raising the question whether other members of the gonococcal FNR regulon had been missed because they also are expressed at a level below the threshold set in this analysis. However, close inspection of the raw data failed to reveal additional candidates that, like *ccp*, were false negatives. Nevertheless, the *ccp *example provides clear evidence that false negative results, like false positive results, can be a problem in microarray analysis.

We recently showed that the list of transcripts differentially expressed in a gonococcal *narP *mutant and its parent is even smaller than the corresponding list for the FNR regulon, and inverted repeat sequences similar to the binding site for *E. coli *NarP were readily identified in only four promoter regions [10]. The *N. gonorrhoeae *NarP is only distantly related to the *E. coli *NarP or NarL (42% and 41.5% sequence identity, respectively), so although gonococcal NarP can recognise and bind to the same inverted repeat sequence as *E. coli *NarP, it is not surprising that it cannot functionally complement *E. coli narXL *or *narQP *mutants. Apart from genes involved in denitrification, only two transcripts encoding proteins of unknown function were regulated by NarP, unlike in *E. coli *in which there is an extensive regulon of genes involved in anaerobic metabolism that are repressed by nitrate-activated NarP [8].

The proposal that NarQ from *N. gonorrhoeae *is a ligand insensitive sensor kinase locked on in its kinase mode is also fully supported by experiments presented in Tables 2 and 3, consistent with the fundamental differences in nitrate and nitrite sensing between the two types of bacteria. *E. coli *NarQ is exquisitely sensitive to nitrate, but two orders of magnitude less sensitive to nitrite [30]. In contrast, gonococcal NarQ is insensitive to both nitrate and nitrite, and induction of *aniA *transcription in the presence of nitrite requires inactivation of the repressor, NsrR, not by nitrite but by the product of nitrite reduction, nitric oxide [10]. As gonococci lack the alternative electron transfer pathways that in *E. coli *are subject to NarP repression and that they are unable to metabolise nitrate, it is entirely consistent that they also lack a nitrate-sensing two-component regulatory system. Consequently, data in Table 2 show that gonococcal NarQ can constitutively phosphorylate *E. coli *NarL or NarP. Conversely, amino acid substitutions that make the P box of *E. coli *NarQ more like that of the gonococcal NarQ simply inactivate signal transduction (Table 3), as had been found in previous detailed site-directed mutagenesis experiments of *E*. *coli *NarX [25-28].

## Conclusion

Both *E. coli *and *N. gonorrhoeae *are Gram-negative human pathogens that show adaptation to, and are able to live in, anaerobic niches as part of their normal colonization-transmission cycles within the host. Both species utilize a common regulator, FNR, in the control of this response. However, this study shows that whereas a wide range of responses and physiological adaptations are coordinated by this FNR in *E. coli*, the adaptations are far fewer and are specifically and primarily focussed upon the immediate metabolic needs for utilising alternate electron acceptors under anaerobic conditions in *N. gonorrhoeae*. As such, while this regulator controls what appears to be a fully integrated response in *E. coli*, in *N. gonorrhoeae *the response is essentially independent of the other physiological changes required for adaptation to anaerobic growth. Furthermore, differences between the ligand sensing and signal transduction capabilities of the *E. coli *and *N. gonorrhoeae *NarQ-NarP proteins were revealed. This illustrates fundamental differences between the way in which environmental responses are controlled and integrated in these two species, and highlights the importance of specific investigations of species with different adaptation strategies.

## Methods

### Strains, plasmids, oligonucleotide primers, and gene identification numbers used in this study

The strains and plasmids used in this study are listed in Table 4. Sequences of oligonucleotide primers are available see Additional file 1. The XNG gene identification numbers used for some genes in this study (those not annotated in the GenBank database) refer to the genome sequence annotation of *N. gonorrhoeae *strain FA1090 that was used in the design of the pan-*Neisseria *microarray [31] and pan-*Neisseria *microarray-v2 [32]. A GBrowse database containing this annotation comparatively presented against other neisserial genome annotations can be found at . COG identifications were made using the NCBI Conserved Domain Search tool [33].

### Growth of *N. gonorrhoeae*

*N. gonorrhoeae *was grown on gonococcal agar plates and in gonococcal broth (GCB) supplied by BD. Solid and liquid media were supplemented with 1 % (v/v) Kellogg's Supplement [34]. For liquid cultures, 2 μl of a stock of *N. gonorrhoeae *was plated onto a gonococcal agar plate and incubated in a candle jar at 37°C for 24 hours. Bacteria from this plate were swabbed onto a second plate and incubated in the same way for a further 16 hours. The entire bacterial growth from this second plate was swabbed into 10 ml of GCB and incubated at 37°C in an orbital shaker at 100 rpm for one hour. This 10 ml pre-culture was then tipped into 50 ml of GCB in a 100 ml conical flask and incubated in the same way. For growth with nitrite, the pre-culture was supplemented with 0.5 mM NaNO_2 _and the flasks were supplemented with 5 mM NaNO_2_.

### Preparation of RNA for microarray experiments

Samples (10 ml) of bacterial culture were mixed with an equal volume of RNAlater (Ambion), the bacteria were pelleted by centrifugation, resuspended in 0.5 ml of RNAlater and stored at 4°C overnight. Bacteria were collected by centrifugation and resuspended in TRIzol (Invitrogen) by vortexing for ten minutes. Chloroform was added, the phases were separated and the aqueous phase was transferred to a clean tube. Crude RNA in the aqueous phase was precipitated with isopropanol and cleaned using an RNeasy kit (QIAGEN). Purified RNA was eluted in RNase-free water with 2 % (v/v) SuperaseIN RNase inhibitor (Ambion).

### cDNA generation, labelling, and microarray hybridisation

Reagents and enzymes for the preparation of materials for microarray hybridisations were sourced from the 3DNA Array 900 MPX kit (Genisphere, PA, USA) unless otherwise stated. One microgram of RNA was reverse transcribed into unlabelled cDNA using SuperScript III reverse transcriptase (Invitrogen) at 42°C for two hours. The cDNA was cleaned using a Clean & Concentrate-5 column (Zymo Research) and poly-T tailed with terminal deoxynucleotidyl transferase. Dye-specific capture sequences were ligated to the poly-T tails and the tagged cDNAs were cleaned using a Clean & Concentrate-5 column. The pan-*Neisseria *microarray v-2 [32], containing probes to *N. gonorrhoeae *and *N. meningitidis *genes, was used for these experiments. Microarray slides were pre-hybridised in 3.5 × SSC, 0.1 % SDS and 10 mg mL^-1 ^BSA for 65°C for 20 minutes, washed with water and isopropanol, dried with an airbrush, and pre-scanned to check for array defects. The capture sequence tagged cDNAs were hybridised onto the microarray slide for 16 h at 60°C in a SlideBooster with the power setting at 25 and a pulse/pause ratio of 3:7. Following the first hybridisation, the slides were washed in 2 × SSC, 0.2 % SDS for 10 min. at 60°C, followed by washes at 2 × SSC and 0.2 × SSC for ten minutes, each at room temperature. The slides were dried with an airbrush and hybridised with the Cy 3 and Cy 5 capture reagents at 55°C for 4 h in a SlideBooster. The slides were again washed in 2 × SSC, 0.2 % SDS (10 min. at 60°C) followed by 10 min. room temperature washes in 2 × SSC and 0.2 × SSC (10 min. at room temperature) and dried with an airbrush. Dried slides were scanned using a ScanArray ExpressHT (Perkin Elmer) using autocalibration. For slides PNA6_29 – PNA6_39 this scanner was unavailable and the image data was collected using a GenePix 4000B (Axon Instruments) and manual calibration.

### Microarray data analysis

Where necessary, scanned microarray images were straightened with ImageViewer (BlueGnome). Images were analysed using BlueFuse for Microarrays (BlueGnome). Spot data were extracted from images and manually flagged to remove artefacts before fusion. Fused data were filtered according to pON value [35]. Spots with pON values less than 0.5 in both channels were excluded to eliminate the bias generated by the inclusion of unhybridized spots in the statistical interpretation of the data, and the data globally adjusted such that the mean rRNA ratio was 1.0. The data were then analysed using BASE. For each pair wise comparison, gene expression median fold-changes were calculated from the biological replicates using the MGH fold-change algorithm, and the Student's t-test was used to assess statistical significance. Since six biological replicates were analysed, a *p *value of 0.01 was used. Genes whose transcript levels did not change consistently (i.e. more or less abundant in the mutant compared to the parental strain) in all the biological replicates in which they were detected for each experiment were discarded. Data were also analysed using a locally prepared implementation of the Cyber-T algorithm within BASE; the results from this analysis is available online at . Total microarray data have been deposited in the ArrayExpress database  with the accession number E-MEXP-726.

### Generation of a chromosomal FNR-3xFLAG fusion in *N. gonorrhoeae*

Codons for a 3x FLAG tag, (DYKDDDDK)3, were linked in-frame to the 3' end of the *fnr *gene on the chromosome of *N. gonorrhoeae *strain F62. Plasmid pGCFNR3 contains the gonococcal *fnr *gene and 500 bp of downstream sequence under the control of the *E. coli fnr *promoter [5]. Inverse PCR, using primers FNRiPCRFwd and FNRiPCRRwd was used to introduce a *Kpn*I restriction site in place of the *fnr *gene stop codon and an *Xho*I site immediately downstream of the *fnr *gene, yielding plasmid pGCFNRi. Sequences of all oligonucleotide primers used in this study are available on-line in Table S1. The kanamycin resistance cassette and the 3x FLAG tag encoded by pSUB11 [36] were amplified by PCR using primers FLAGFwd and FLAGRwd, which introduced *Kpn*I and *Xho*I sites at either end of the resultant fragment. The Kan^R^-3x FLAG fragment and pGCFNRi were digested with *Kpn*I and *Xho*I, the plasmid fragment was dephosphorylated with calf alkaline phosphatase, and the two fragments were ligated to form plasmid pGCFNR-FLAG. Western blotting was used to show that pGCFNR-FLAG expressed a 30 kDa FLAG-tagged protein in *E. coli*, corresponding to the gonococcal FNR. To transfer the *fnr*-3x FLAG-Kan^R ^fragment into *N. gonorrhoeae*, pGCFNR-FLAG was digested with *Hin*dIII and *Bam*HI and the 3.5 kb *fnr*-3x FLAG-Kan^R ^fragment was purified by phenol chloroform extraction and ethanol precipitation. Piliated *N. gonorrhoeae *strain F62 was transformed with this linear DNA fragment, which recombined with the *fnr *locus on the gonococcal chromosome yielding strain JCGC502. To confirm that the FLAG-tagged FNR protein was still functional and able to activate *aniA *expression, the ability of strain JCGC502 to utilise nitrite was determined. Cultures of JCGC502 were grown microaerobically in the presence and absence of nitrite and optical densities were measured at hourly intervals. Strain JCGC502 grew exponentially and respired nitrite, therefore AniA was expressed and the FLAG-tagged FNR protein was functional. In addition, samples taken from the cultures at hourly intervals were probed by Western blotting to determine the quantity of FNR-3xFLAG present (see Additional file 2). No significant differences were observed either over the course of the growth curves or between cultures grown in the presence or absence of nitrite. These data confirm that gonococcal FNR activity is likely to be modulated by oxygen in a manner similar to the *E. coli *FNR protein, rather than expression level, as it the case of some other CRP-FNR superfamily members such as *Bradyrhizobium japonicum *FixK_2 _[37].

### Western blotting

Gonococcal proteins separated by Tris/Tricine SDS-PAGE using a 15% polyacrylamide gel were blotted onto a PVDF membrane and FLAG-tagged FNR protein was detected using anti-FLAG monoclonal antibodies (Sigma) and the ECL-Plus chemiluminescence detection system (GE Healthcare Life Sciences).

### Chromatin Immunoprecipitation

Interactions between FNR and promoter DNA were studied *in vivo *by Chromatin Immunoprecipitation (ChIP) as described by Grainger *et al*. [38]. *N. gonorrhoeae *strain JCGC502 was grown microaerobically with or without 5 mM NaNO_2 _to late exponential phase. Protein-DNA crosslinking, chromatin preparation, and immunoprecipitations were as described previously except that the tagged protein was immunoprecipitated with anti-FLAG monoclonal antibodies (Sigma) for 16 h at 4°C. The concentration of immunoprecipitated promoter fragments was measured using quantitative real time PCR [39]. Primers for each promoter were designed using PrimerExpress (Applied Biosystems) and are listed in Table S1. The promoter of the *hpt *(NG2035) gene, which is not regulated by FNR and is not preceded by an FNR binding site, was a negative control used to normalise the data. Promoter fragments enriched by 60% or more in at least two independent ChIP experiments, relative to the *hpt *promoter fragment, were scored positive.

### Construction of *E. coli *strains

The *narXL *genes were deleted from *E. coli *strain JCB386 using the gene-replacement method [36]. Primers EcnarXp1 and EcnarLp2 were used to amplify the chloramphenicol acetyltransferase gene from plasmid pKD3 [40] resulting in a *cat *cassette flanked by DNA with sequence homology to upstream and downstream of the *narXL *genes. This linear DNA fragment was electroporated into strain *E. coli *JCB386 containing pKD46, encoding the λ Red recombinase, which mediated recombination of the chloramphenicol resistance cassette into the *E. coli *chromosome and replacement of the *narXL *genes with the *cat *gene. Transformation of the *narXL*::chl^R ^strain with pCP20, encoding FLP recombinase, resulted in loss of the chloramphenicol resistance gene and creation of an unmarked *narXL *deletion in strain JCB3861. The *narQ *deletion was generated by the same method, using primers EcnarQp1 and EcnarQp2, resulting in strain JCB3862 (*narXL narQ*). The *narP *deletion was transferred using P1 transduction; strain JCB3862 was transduced with bacteriophage P1 that had been propagated on *E. coli *strain JCB3875 which carries a *narP*::chl^R ^mutation [17], generating strain JCB3863 (*narXL narQ narP*). Strain JCB391 was generated by successive transduction of the *narXL*::chl^R ^and *narQ*::chl^R ^mutations into strain RV followed by removal of the antibiotic resistance cassettes using pCP20. The *pcnB*::kan^R ^mutation, effectively reducing the plasmid copy number to one, was transferred from strain RP7974 [41].

### Construction of plasmids expressing gonococcal and *E. coli narQP*

The gonococcal *narQP *genes were amplified from chromosomal DNA by PCR using primers NgNarQPNcoI and NgNarQPBamHI, generating a fragment with *Nco*I and *Bam*HI sites at each end. The resultant PCR product and pGCFNR3 were both digested with *Nco*I and *Bam*HI and ligated, yielding pGCNarQP. For plasmid pBADgcQ, the gonococcal *narQ *gene was cloned into the arabinose-inducible pBAD *myc*-hisA overexpression vector using primers NgNarQNcoI and NgNarQHindIII to generate an *Nco*I – *Hin*dIII *narQ *fragment, which was ligated into *Nco*I -*Hin*dIIIdigested pBAD *myc*-hisA (Invitrogen). Similarly, pBADecQ contained the *E. coli narQ *gene cloned into pBAD *myc*-hisA. Primers EcNarQ *Nco*I and EcNarQ *Bam*HI were used to generate a *Nco*I-*Bam*HI *E. coli narQ *fragment, which was cloned into pBAD *myc*-hisA.

The Quikchange site-directed mutagenesis system (Stratagene) was used to generate specific mutations in the P-box region of the *E. coli narQ *gene using primers listed in Table S1: Primer pair SDM1 R-K FWD & RVS were used to generate substitution R54K; pair SDM2 NI-EE FWD & RVS substitutions N48E & I49E; SDM3 DAEA-AASV FWD & REV substitutions D43A E45S & A46V ; SDM4 DAEA-AASV FWD & RVS substitutions D43A E45S & A46V; and SDM5 SS-NA FWD & RVS substitutions S52N & S57A. Substitutions were combined by stepwise mutagenesis in plasmids pRNW18-34 as listed in Table 4.

### β-galactosidase assay

*E. coli *was grown at 37°C or 30°C in LB (Luria-Bertani) medium with 0.4 % glucose or in minimal medium [42] supplemented with 40 mM sodium fumarate, 10 % LB and 0.4 % glycerol. Where stated, cultures were supplemented with 20 mM NaNO_3 _or 2.5 mM NaNO_2_. Two ml aliquots of bacterial cultures were lysed by the addition of 30 μl each toluene and 2 % (w/v) sodium deoxycholate and aerated at 30°C for 20 minutes. Lysates were assayed for β-galactosidase activity as previously described [15].

### Sequence pattern searching

Potential FNR binding sites were located in promoter regions using Findpatterns in the GCG suite (Accelrys, Cambridge, UK) using the consensus *E. coli *FNR binding site, TTGATNNNNATCAA, to search the gonococcal DNA sequences.

## Abbreviations

ChIP, Chromatin Immunoprecipitation; FNR, regulator of fumarate and nitrate reduction; NsrR, nitrosative stress response regulator; qRT-PCR, quantitative reverse transcriptase polymerase chain reaction; HAMP, histidine kinase, adenylate cyclase, methyl-accepting protein and phosphotransferase domain; TMII, second transmembrane region; RNS, Reactive Nitrogen Species; ROS, Reactive Oxygen Species; ; OMP, outer membrane protein; CDS, coding sequence.

## Authors' contributions

RW, TWO and JAC designed the experimental work. RW and TWO performed the experimental work and analysed the data. SM developed and implemented the use of GBrowse for the display of the microarray data. LASS & NJS designed and manufactured the PNAv2 microarrays and supervised the microarray-based experiments and analysis. RW, TWO, LASS, HS, JAC and NJS drafted the manuscript. All authors read and approved the final manuscript.

## Supplementary Material

Additional file 1 Primers used in this study. Primer sequences used for construction of plasmids and strains, mutagenesis, and quantitative real-time PCR Click here for file

Additional file 2 Figure of growth curves of JCGC501 and anti FNR-3xFLAG Western blots. Figure showing: A. Growth characteristics of strain JCGC501, carrying a chromosomal *fnr*-3xFLAG fusion. Strain JCGC501 was grown microaerobically in the presence or absence of 5 mM NaNO_2_. B. Western blotting shows that the quantity of FNR-3xFLAG protein remains constant through the growth cycle. Samples were taken from the above growth curve at hourly intervals, separated by SDS-PAGE, blotted onto PVDF membrane and FNR-3xFLAG protein was detected using anti-FLAG antibodies and ECF-Plus chemiluminescent labelling. Click here for file

## Figures and Tables

**Table 1:** Genes differentially expressed in *fnr*^+ ^and *fnr *strains of *N. gonorrhoeae*.

***fnr***^**+**^**+ NO**^**2-**^**/*fnr***		***fnr***^**+**^** - NO**^**2-**^**/*fnr***							
				**Gene number**	**Gene name**		**FNR binding site upstream**		**ChIP enrichment**
						**Product**	**Sequence**	**Location**	
Transcripts more abundant in the **Ratio t-test Ratio t-test ***fnr*^+ ^strain									

**46.30**	**1.1 × 10 *p *value**^**-5**^	**73.06**	**7.3 × 10 *p *value**^**-6**^	NMB1205		Transcript of unknown function	**TTGAT**TTGA**ATCAA**	**41.5**	Yes
**12.27**	**3.2 × 10**^**-4**^	**6.60**	**7.3 × 10**^**-5**^	NGO1688	*ompU*	Putative iron uptake OMP	none		Yes
**6.02**	**1.9 × 10**^**-5**^	**3.56**	**8.2 × 10**^**-4**^	NGO1276	*aniA*	Nitrite reductase	**TTGAC**TTAA**ATTAA**	**88.5**	Yes §
**4.57**	**4.8 × 10**^**-4**^	**3.52**	**3.2 × 10**^**-5**^	NGO1215		Conserved hypothetical protein (COG2847)	**TTAAT**CTAC**ATCAA**	**73.5**	NE
**2.31**	**2.1 × 10**^**-3**^	**2.88**	**5.2 × 10**^**-5**^	NGO0546	*res*	Type III restriction-modification system EcoPI enzyme	**TTGAA**TGTG**ATTAT**	**106.5**	NE
**4.21**	**1.9 × 10**^**-3**^	ND		XNG1301	*nosR*	Regulatory protein	**TTGAT**TTGC**ATCAA**	**20.5**	NT
**2.85**	**3.1 × 10**^**-3**^	0.72	0.06	NGO1621		Putative phage associated protein	none		NT
**2.75**	**1.0 × 10**^**-3**^	0.83	0.28	NGO1622		Putative phage associated protein	none		NT
**2.70**	**4.1 × 10**^**-3**^	0.86	0.47	NGO0472		Putative phage associated protein	none		NT
**2.63**	**1.7 × 10**^**-4**^	1.00	0.51	NGO1455		Putative NRAMP family Manganese/Iron transporter	none		NE
**2.56**	**5.1 × 10**^**-3**^	2.80	0.05	NGO0602		Putative MerR family transcriptional regulator	**TTGTT**TCAT**TTCCA**	**130.5**	NT
**2.20**	**9.5 × 10**^**-3**^	1.21	0.16	NGO0087		Hypothetical protein	none		NT
**2.10**	**2.0 × 10**^**-3**^	1.02	0.83	NGO0473		*Neisseria *specific protein.	**GTGAT**TCTG**ACAAA**	**199.5**	NE
**2.01**	**1.4 × 10**^**-3**^	1.00	0.71	NGO1615		Putative phage associated protein	**TTCAG**CTGC**TTCAA** **CTGAT**CAGC**CTCAT**	**70.5** **25.5**	NT
									
Transcripts more abundant in the *fnr *strain									
**0.14**	**9.6 × 10**^**-6**^	**0.18**	**8.1 × 10**^**-6**^	NGO1716		Putative phosphotransferase	**TTGAG**TGAA**AATAA**	**85.5**	Yes
**0.29**	**7.3 × 10**^**-3**^	1.23	0.39	NGO0340	*cysK*	Cysteine synthetase	none		Yes
**0.47**	**3.4 × 10**^**-4**^	1.47	0.04	NGO0374	*glnQ*	Amino acid ABC transporter	none		NT
0.37	0.12	**0.19**	**8.4 × 10**^**-6**^	NGO0653	*dnrN*	Putative NO- response protein	**ATGTT**CAAA**ACCAA**	**36.5**	NE
0.60	0.84	**0.28**	**2.9 × 10**^**-3**^	NGO1275	*norB*	Nitric oxide reductase	**TGAAT**TTTA**AACAA**	**33.5**	Yes §
0.91	0.31	**0.49**	**2.6 × 10**^**-4**^	NGO1428		Short *Neisseria *specific protein	none		NE

The ratio of transcript intensity and Student's t-test *p *value are shown for each gene in each comparison; *fnr*^+ ^+ NO_2 _^-^/*fnr *and *fnr*^+ ^- NO_2 _^-^/*fnr*. Predicted FNR binding sites with at most 3 mismatches to the consensus *E. coli *FNR binding site, TTGATNNNNATCAA, located up to 200 bases upstream of the translation start of the gene, are displayed. Note that positions 33.5, 88.5 etc indicate that the central bases of the inverted repeat sequence are located between bases 33 and 34, or between bases 88 and 89, upstream from the translation start. A ChIP enrichment of 60% or more is scored positive. ND, No data for this gene; NE, no enrichment; NT, not tested by ChIP. § The *aniA *and *norB *promoters are divergent; ChIP detected FNR binding to the *aniA-norB *region but cannot be used to resolve the two promoters.

**Table 2:** Complementation of *E. coli narXLQP *mutations by gonococcal NarQ and NarQP.

Host strain	Host genotype	Plasmid	Reporter	β-galactosidase activity		
				Ø_2_	+ NO_2 _^-^	+ NO_3 _^-^
A. Complementation of a *narXLQP *mutation						
JCB386		none	*nirB*::*lacZ*	120	650	1200
JCB3861	*narXL*	none	*nirB*::*lacZ*	80	130	570
JCB3863	*narXLQP*	none	*nirB*::*lacZ*	49	39	61
JCB3863	*narXLQP*	pGCNarQP	*nirB*::*lacZ*	64	67	81

B. Phosphorylation of *E. coli *NarP by gonococcal NarQ						
JCB391	*narXLQ*	none	*napF*::*lacZ*	130	110	120
JCB391	*narXLQ*	pBADgcQ	*napF*::*lacZ*	1630	1390	1350

C. Phosphorylation of *E. coli *NarL by gonococcal NarQ						
JCB12	*narXQ*	none	*frdA::lacZ*	2830	2070	2570
JCB12	*narXQ*	pBADgcQ	*frdA::lacZ*	780	650	820

*E. coli *transformed with the stated plasmids was grown anaerobically in LB in A. and B. and in minimal medium in C. in the absence or presence of 2.5 mM NaNO_2 _or 20 mM NaNO_3 _and the β-galactosidase activities determined.

**Table 3:** P-box substitutions in *E. coli *NarQ.

Plasmid	Substitutions	P-box sequence	Phenotype
pBADEcQ	*E. coli *NarQ P-box	DAEAINIAGSLRMQSYRL	Nitrate and nitrite sensing
pRNW18	R54K	DAEAINIAGSL**K**MQSYRL	Ligand insensitive
pRNW19	N48E I49E	DAEAI**EE**AGSLRMQSYRL	Ligand insensitive
pRNW20	N48E I49E R54K	DAEAI**EE**AGSL**K**MQSYRL	Ligand insensitive
pRNW29	D43A E45S A46V N48E I49E	**A**A**SV**I**EE**AGSLRMQSYRL	Ligand insensitive
pRNW30	D43A E45S A46V	**A**A**SV**INIAGSLRMQSYRL	Ligand insensitive
pRNW31	D43A E45S A46V R54K	**A**A**SV**INIAGSL**K**MQSYRL	Ligand insensitive
pRNW32	D43A E45S A46V N48E I49E R54K	**A**A**SV**I**EE**AGSL**K**MQSYRL	Ligand insensitive
pRNW33	D43A E45S A46V S52N S57A	**A**A**SV**INIAG**N**LRMQ**A**YRL	Ligand insensitive
pRNW34	D43A E45S A46V N48E I49E S52N R54K S57A	**A**A**SV**I**EE**AG**N**L**K**MQ**A**YRL	Ligand insensitive

*E. coli *strains JCB391 (*narXL narQ*) or JCB12 (*narXL narQ frdA::lacZ*) transformed with pBADecQ expressing wild-type or mutated NarQ proteins were grown anaerobically in LB in the absence or presence of 20 mM NaNO_3 _and the β-galactosidase activities determined. Mutations in the P box are shown in bold type. Wild-type NarQ was able to phosphorylate NarP in the presence of nitrate, activating expression of *nirB *and repressing *frdA*. Mutated NarQ proteins were inactive.

**Table 4:** Strains and plasmids used in this study.

**Strain**	**Description**	**Source**
***N. gonorrhoeae *strains**		
F62	Parental strain	Laboratory stocks
RUG7001	F62 *proAB *p*aniA::lacZ*	[43]
RUG7022	F62 *proAB *p*aniA::lacZ fnr*	[6]
JCGC201	F62 *proAB *p*ccp::lacZ*	[5]
JCGC202	F62 *proAB *p*ccp::lacZ fnr*	[5]
JCGC502	F62 *fnr-*3xFLAG Kan^R^	This work
***E. coli *strains**		
RV	Parental strain	Laboratory stocks
JCB386	Δ (*nirB-cycG*) *pnirB::lacZ*	[44]
JCB3861	JCB386 *narXL*	This work
JCB3862	JCB386 *narXL narQ*	This work
JCB3863	JCB386 *narXL narQ narP*	This work
JCB391	RV *narXL narQ pcnB*	This work
JCB12	RV *narX narQ frdA::lacZ*	[10]
**Plasmids**		
pGCFNR3	Gonococcal *fnr*, under the regulation of the *E. coli fnr *promoter, cloned into pBR322.	[5]
pGCFNRi	pGCFNR3 with *Kpn*I and *Xho*I restriction sites engineered downstream of the *fnr *gene	This work
pGCFNR-FLAG	pGCFNR3 with a 3xFLAG tag and Kanamycin resistance cassette inserted downstream of the *fnr *gene	This work
pSUB11	Epitope tagging plasmid carrying 3xFLAG tag and kanamycin resistance cassette.	[36]
pGCNarQP	Gonococcal *narQP *genes under the control of the *E. coli fnr *promoter cloned into pBR322.	This work
pBADgcQ	Gonococcal *narQ *gene under the control of the *araBAD *promoter cloned into pBAD *myc*-His A.	This work
pBADecQ	*E. coli narQ *gene under the control of the *araBAD *promoter cloned into pBAD *myc*-His A	This work
pRNW18	pBADEcQ containing R54K substitution in *narQ*	This work
pRNW19	pBADEcQ containing N48E I49E substitution in *narQ*	This work
pRNW20	pBADEcQ containing N48E I49E R54K substitution in *narQ*	This work
pRNW29	pBADEcQ containing D43A E45S A46V N48E I49E substitution in *narQ*	This work
pRNW30	pBADEcQ containing D43A E45S A46V substitution in *narQ*	This work
pRNW31	pBADEcQ containing D43A E45S A46V R54K substitution in *narQ*	This work
pRNW32	pBADEcQ containing D43A E45S A46V N48E I49E R54K substitution in *narQ*	This work
pRNW33	pBADEcQ containing D43A E45S A46V S52N S57A substitution in *narQ*	This work
pRNW34	pBADEcQ containing D43A E45S A46V N48E I49E S52N R54K S57A substitution in *narQ*	This work
p7150	*E. coli nirB *promoter cloned into the *lacZ *reporter vector pRW50.	[19]
pRNW15	*E. coli napF *promoter cloned into the *lacZ *reporter vector pRW50.	This work
