# Supplementary material for: The small FNR regulon of Neisseria gonorrhoeae: comparison with the larger Escherichia coli FNR regulon and interaction with the NarQ-NarP regulon
Source: BMC Genomics. 2007 Jan 29;8:35. doi: 10.1186/1471-2164-8-35 (PMC1802743; doi:10.1186/1471-2164-8-35)
Supplement: Additional file 1 — Primers used in this study. Primer sequences used for construction of plasmids and strains, mutagenesis, and quantitative real-time PCR [file 1471-2164-8-35-S1.doc]

| Primer name | Function | Nucleotide sequence 5’ – 3’ |
| --- | --- | --- |
| *Primers for construction of plasmids and strains (introduced restriction sites are marked in* ***bold****)* | |  |
| NgNarQPNcoI | Amplification of gonococcal *narQP* genes | ATATA**CCATGG**TACTGCCAACCCGATTTTCAGACGG |
| NgNarQPBamHI | Amplification of gonococcal *narQP* genes | AATAA**GGATCC**TAAACTGCCTAAGCCATTCCGTCCG |
| NgNarQNcoI | Amplification of gonococcal *narQ* gene | ATATA**CCATGG**TACTGCCAACCCGATTTTCAGACGG |
| NgNarQHindIII | Amplification of gonococcal *narQ* gene | ATC**AAGCTT**TCATGGTAGGCTTTCTTTGGGTGCG |
| EcNarQNcoI | Amplification of *E. coli narQP* gene | ATATAA**CCATGG**TTGTTAAACGACCCGTCTCGGCC |
| EcNarQHindIII | Amplification of *E. coli narQP* gene | AAA**AAGCTT**TTACATTAACTGACTTTCCTCACCCTC |
| FNRiPCRFwd | Generation of *Kpn*I & *Xho*I sites in pGCFNR3 | ATAA**GGTACC**ACAATA**CTCGAG**CGAACATTTCAGACGGC |
| FNRiPCRRwd | Generation of *Kpn*I & *Xho*I sites in pGCFNR3 | AATA**GGTACC**AATGGCGTGCGAACAACC |
| FLAGFwd | Amplification of 3xFLAG Kan^R^ cassette from pSUB11 | ATAT**GGTACC**GACTACAAAGACCATGACGG |
| FLAGRwd | Amplification of 3xFLAG Kan^R^ cassette from pSUB11 | TGAC**GAGCTC**TATACTTATAGGAGGAATCAAGG |
| EcnarXp1 | Chromosomal deletion of *E. coli narXL* genes | AGGTTATTGCTCATTTAAAGCCTGAAGGAAGAGGTTTACTGTGTAGGCTGGAGCTGCTTC |
| EcnarLp2 | Chromosomal deletion of *E. coli narXL* genes | GATGCATTGTCAAACGACGAACTGCGCTGGGAACCGTAACATATGAATATCCTCCTTAG |
| EcnarQp1 | Chromosomal deletion of *E. coli narQ* gene | GAACTGGAACATTAATGATTTTTTGTGGAGAAGACGCGTTGTGTAGGCTGGAGCTGCTTC |
| EcnarQp2 | Chromosomal deletion of *E. coli narQ* gene | GAACCTTAAGTGCAAGTCTTCTTTGGTCAGTAGGAGGCACATATGAATATCCTCCTTAG |
|  |  |  |
| *Mutageneic primers (mutations are marked in* ***bold****)* | | |
| SDM1 R-K FWD | Generation of substitution R54K in *E. coli* NarQ | GCCGGATCGCTG**AAG**ATGCAGAGTTACCGC |
| SDM1 R-K RVS | Generation of substitution R54K in *E. coli* NarQ | GCGGTAACTCTGCAT**CTT**CAGCGATCCGGC |
| SDM2 NI-EE FWD | Generation of substitutions N48E & I49E in *E. coli* NarQ | GCTGAGGCTATC**GAAGAG**GCCGGATCGCTG |
| SDM2 NI-EE RVS | Generation of substitutions N48E & I49E in *E. coli* NarQ | CAGCGATCCGGC**CTCTTC**GATAGCCTCAGC |
| SDM3 DAEA-AASV FWD | Generation of substitutions D43A E45S & A46V in *E. coli* NarQ | GCAGTTTGCGC**GCCGCCTCGGTC**ATCGAAGAGGCG |
| SDM3 DAEA-AASV RVS | Generation of substitutions D43A E45S & A46V in *E. coli* NarQ | CGCCTCTTCGAT**GACCGAGGCGGC**GCGCAAACTGC |
| SDM4 DAEA-AASV FWD | Generation of substitutions D43A E45S & A46V in *E. coli* NarQ | GCAGTTTGCGC**GCCGCCTCGGTC**ATCAATATTGCC |
| SDM4 DAEA-AASV RVS | Generation of substitutions D43A E45S & A46V in *E. coli* NarQ | GGCAATATTGAT**GACCGAGGCGGC**GCGCAAACTGC |
| SDM5 SS-NA FWD | Generation of substitutions S52N & S57A in *E. coli* NarQ | GAAGAGGCCGGA**AAT**CTGAAGATGCAG**GCA**TACCGCCTGGGC |
| SDM5 SS-NA RVS | Generation of substitutions S52N & S57A in *E. coli* NarQ | GCCCAGGCGGTA**TGC**CTGCATCTTCAG**ATT**TCCGGCCTCTTC |
| *Primers for quantitative real time PCR detection of promoter regions* | | |
| aniA_266F | Detection of *aniA* promoter in ChIP | CCGCGACTATCCTGCCAAAGTA |
| aniA_367R | Detection of *aniA* promoter in ChIP | TCGCCGTCAAATGTCCAGTAGC |
| B1205S region_66F | Detection of B1205S promoter in ChIP | GATCGGCAAAATCGGTTTGC |
| B1205S region_171R | Detection of B1205S promoter in ChIP | TATGCCGTCTGATGATGCAGG |
| ccp_381F | Detection of *ccp* promoter in ChIP | TGGCGGCTACGGTAATGGTTA |
| ccp_516R | Detection of *ccp* promoter in ChIP | CCCAAATGCGATAAGAGCGTC |
| cysK_60F | Detection of *cysK* promoter in ChIP | TTTATGCGCTGAAGCCGGTTT |
| cysK_207R | Detection of *cysK* promoter in ChIP | ATCAACATCGCCGCCAACA |
| dnrN_102F | Detection of *dnrN* promoter in ChIP | TGGCTTCGTTGTCGTCGATGT |
| dnrN_202R | Detection of *dnrN* promoter in ChIP | CGGCCCCTGCAAAATGATT |
| hpt_110F | Detection of *hpt* promoter in ChIP | AAGGACAAAGACCGCGACAATC |
| hpt_221R | Detection of *hpt* promoter in ChIP | TACGCACGCGCACAAATGT |
| NGO0473_198F | Detection of NGO0473 promoter in ChIP | ATTCTGACAAAAGCGCGCC |
| NGO0473_299R | Detection of NGO0473 promoter in ChIP | AATTTGGCTGCCGTCTTGC |
| NGO0546_208F | Detection of NG0546 promoter in ChIP | TCGGCAGCAATATGGCAAG |
| NGO0546_379R | Detection of NGO0546 promoter in ChIP | TTGTTGTATCGTCCTTCCCCC |
| NGO1215_165F | Detection of NGO1215 promoter in ChIP | GAAAAACACGAGCTGGCCAAA |
| NGO1215_277R | Detection of NGO1215 promoter in ChIP | CAAGCGTTTTACCCGAACAGG |
| NGO1428_79F | Detection of NGO1428 promoter in ChIP | ATGACCGACAAACTGCCCGTA |
| NGO1428_244R | Detection of NGO1428 promoter in ChIP | TATCGATTGGCTGCTGACAGG |
| NGO1455_363F | Detection of NGO1455 promoter in ChIP | GGCGGTATCCTGTTTTTGAAGA |
| NGO1455_466R | Detection of NGO1455 promoter in ChIP | ACAGAGCCGCATATTCGGACA |
| NGO1688_171F | Detection of NGO1688 promoter in ChIP | ATGTTTTGACACACAGGCGGC |
| NGO1688_274R | Detection of NGO1688 promoter in ChIP | CCGGTTTCATCATTATGCCCC |
| NGO1716_138F | Detection of NGO1716 promoter in ChIP | TCGTTACTGCAAAACAGGCAGG |
| NGO1716_285R | Detection of NGO1716 promoter in ChIP | TCTGTTTCGGCACGCATTG |
