# Supplementary figures and images for: The small FNR regulon of Neisseria gonorrhoeae: comparison with the larger Escherichia coli FNR regulon and interaction with the NarQ-NarP regulon
Source: BMC Genomics. 2007 Jan 29;8:35. doi: 10.1186/1471-2164-8-35 (PMC1802743; doi:10.1186/1471-2164-8-35)

**A**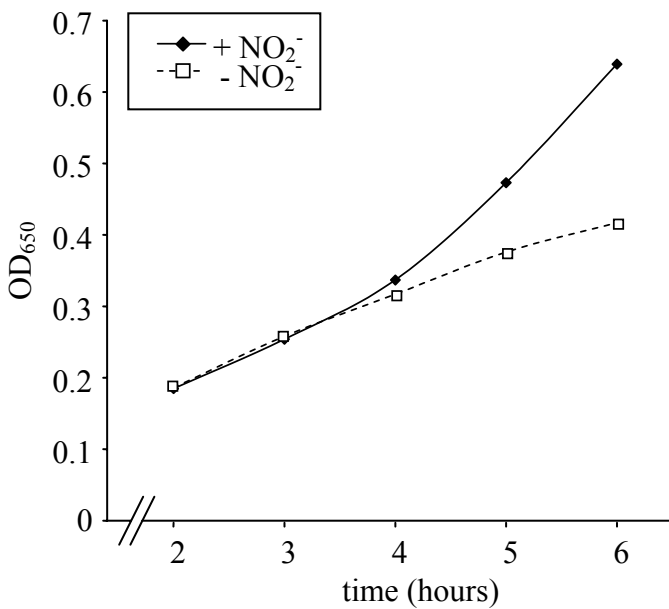**B**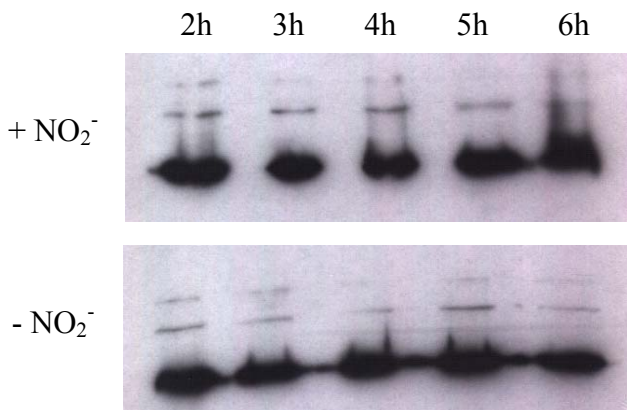

Supplement: Additional file 2 — Figure of growth curves of JCGC501 and anti FNR-3xFLAG Western blots. Figure showing: A. Growth characteristics of strain JCGC501, carrying a chromosomal fnr-3xFLAG fusion. Strain JCGC501 was grown microaerobically in the presence or absence of 5 mM NaNO2. B. Western blotting shows that the quantity of FNR-3xFLAG protein remains constant through the growth cycle. Samples were taken from the above growth curve at hourly intervals, separated by SDS-PAGE, blotted onto PVDF membrane and FNR-3xFLAG protein was detected using anti-FLAG antibodies and ECF-Plus chemiluminescent labelling. [file 1471-2164-8-35-S2.pdf]
